# It’s all in the combination: decoding the epigenome for cancer research and diagnostics

**DOI:** 10.1016/j.gde.2022.101899

**Published:** 2022-04

**Authors:** Noa Furth, Efrat Shema

**Affiliations:** Department of Biological Regulation, Weizmann Institute of Science, Rehovot 76100, Israel

## Abstract

Genome regulation is governed by the dynamics of chromatin modifications. The extensive and diverse array of DNA and histone modifications allow multiple elements to act combinatorically and direct tissue-specific and cell-specific outcomes. Yet, our ability to elucidate these complex combinations and link them to normal genome regulation, as well as understand their deregulation in cancer, has been hindered by the lack of suitable technologies. Here, we describe recent findings indicating the importance of the combinatorial epigenome, and novel methodologies to measure and characterize these combinations. These complementary methods span multiple disciplines, providing a means to decode epigenetic combinations and link them to biological outcomes. Finally, we discuss the promise of harnessing the rich combinatorial epigenetic information to improve cancer diagnostics and monitoring.

**Current Opinion in Genetics & Development** 2022, **73**:101899

This review comes from a themed issue on **Cancer genomics**

Edited by **Luciano di Croce** and **Jane Skok**

For complete overview of the section, please refer to the article collection, “Cancer Genomics”

Available online 25th January 2022

**https://doi.org/10.1016/j.gde.2022.101899**

0959-437X/© 2022 The Author(s). Published by Elsevier Ltd. This is an open access article under the CC BY license (http://creativecommons.org/licenses/by/4.0/).

Cell-type specific chromatin organization, governed by transcription factors (TFs) and chromatin regulators, provides the means for diverse phenotypes arising from identical genetic information [1]. At the basis of the chromatin structure are the nucleosomes, in which the double-stranded DNA is wrapped around the histone octamer. Both the DNA and the four core histone proteins are extensively and dynamically modified by various covalent modifications, affecting multiple aspects of chromatin homeostasis [2]. Histone modifications regulate chromatin packing and 3D conformation, thus controlling access of DNA-binding proteins [3,4,5^•^,^6^]. Of note, this ability is not restricted to polar modifications such as acetylation; mono-methylation on H3 lysine 20 was recently shown to regulate chromatin folding [3]. Furthermore, methylation of H3 lysine 9 promotes the formation of liquid–liquid phase separation, thus regulating chromatin compartmentalization and genome function [7]. Finally, numerous examples exist for histone and DNA modifications acting as unique docking sites for the binding or release of downstream effector proteins [3,8].

The importance of the epigenetic network is reflected in the vital roles it plays in cellular differentiation and its recurrent deregulation in cancer [9,10]. While more than 50% of human cancers harbor mutations in enzymes that are involved in chromatin organization [10], the molecular mechanisms by which cancer cells hijack the epigenetic machinery varies between tumor types and strongly associate with the developmental origins of the different tumors. Chromatin remodelers such as the SWI/SNF complex, as well as central histone modifiers such as PRC1/2 and COMPASS, are recurrently de-regulated in tumors [11, 12, 13], resulting in profound changes to the epigenetic landscape that supports tumorigenic transcriptional programs. Recurrent mutations in the genes encoding the core histones have also been linked to specific tumors [14]. While the mutations appear either in the globular domain or the extensively modified histone tails, the resulting mutant histones, termed ‘oncohistones’, are incorporated into the nucleosome complex. Interestingly, despite the low prevalence of oncohistones in the genome (due to multiple alleles encoding the wt histone), these mutations profoundly affect the epigenome and transcriptome of these cancers [15^•^]. Finally, tumor cells acquire altered DNA methylation (5mC) and hydroxymethylation (5hmC) states due to direct mutations or altered activity of the DNA methylation machinery, which may further alter the histone code and transcription, to maintain the pro-tumorigenic cellular state [16].

## The combinatorial epigenetic code is key in mediating specific chromatin responses

The variety of substrates (histone, DNA and RNA), large number of modified residues and their dynamic nature, support the hypothesis that their functional outcome depends on specific combinations of marks. Indeed, distinct transcriptional output depends on the context and dynamics of multiple histone modifications [17]. Changes in the combinatorial chromatin code confer phenotypic plasticity, which facilitates the ability of cancer cells to cope with hostile environments such as those encountered during metastasis spread or treatment with anti-cancer drugs [18].

**Figure 1:**
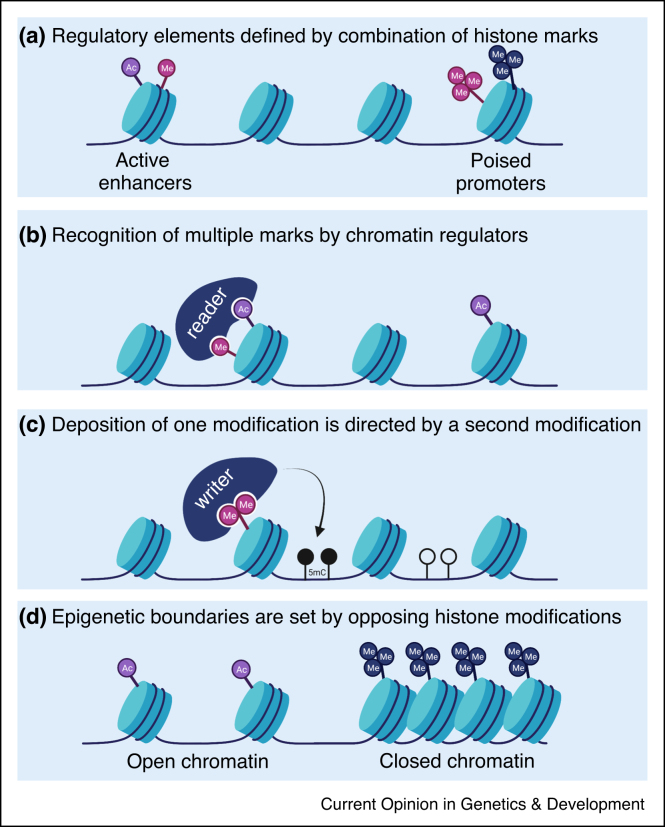
Multiple modes of regulation by combinatorial chromatin patterns. **(a)** Distinct combinations of histone marks specify regulatory elements in the genome, such as promoters and enhancers. **(b)** Chromatin-associated protein complexes associate with multiple different marks, generating specificity in their genomic recruitment and activity. **(c)** Epigenetic writers are directed by specific marks, thus producing combinatorial patterns consisting of different modifications. For example, the DNA methyltrasferase DNMT3A preferentially binds and methylates genomic regions marked by H3K36me2 [24]. **(d)** Different epigenetic pathways drive opposing outcomes, generating boundaries between genomic regions marked by different modifications. For instance, the balance between methylation and acetylation of H3 lysine 27 maintains separation between silenced and transcribed genomic regions [42,43^•^].

The interaction between epigenetic pathways spans multiple nodes of regulation (Figure 1). Chromatin-associated protein complexes frequently contain a combination of several readers specific for distinct histone marks, and many readers associate with a substantial stretch of the histone tail, allowing for the sensing of multiple marks [19]. Mashtalir *et al.* recently reinforced this notion by providing evidence for the additive inhibitory effect of H3 lysine 4 trimethylation (H3K4me3) and H4 poly-acetylation on SWI/SNF remodeling activity [20^••^]. Similarly, the NURF chromatin remodeling complex subunit BPTF selectively binds to nucleosomes marked with both H3K4me3 and H4K16ac [21]. This combinatorial regulation is not restricted to remodeling complexes and is seen for other chromatin binding proteins. In the DNA damage response, the recruitment of 53BP1 following double-strand breaks is dependent on the combined pattern of ubiquitination of H2A on lysine 15 (H2AK15ub) and histone H4 lysine 20 dimethylation (H3K20me2) [8]. The transcriptional repressor MeCP2 is recruited to chromatin to regulate gene expression by recognizing both DNA methylation at CpG islands and tri-methylation of H3 lysine 27 (H3K27me3) [22]. Furthermore, a computational approach has recently revealed multiple combinatorial histone modification patterns affecting alternative splicing in different cellular contexts [23]. Although further functional and biochemical studies are in place, this study strongly suggests that different RNA binding proteins are specifically recruited to combinatorial chromatin signatures to regulate specific biological outcomes.

Epigenetic writers may rely on certain marks to direct the localized deposition of a specific modification. The DNA methyltransferase 3A (DNMT3A) is recruited to intergenic chromatin regions marked by H3K36me2, and mutations in the PWWP recognition domain may shift the recruitment and DNA methylation activity toward chromatin regions marked by H2AK119Ub [24,25^••^]. In addition, the H3K36me3 mark recruits METTL14, a crucial component of the m6A methyltransferase complex, to facilitate the deposition of the RNA modification m6A [26]. Thus, distinct histone modifications dictate the deposition patterns of other DNA and RNA modifications, resulting in combinatorial epigenetic patterns on chromatin and newly synthesized RNA. Similarly, histone methyltransferases are recruited to distinct genomic locations by specific marks; the activity of the histone H3 lysine 79 methyltransferase, Dot1, is allosterically stimulated by both H4K16ac and H2B ubiquitination [27]. Furthermore, the H3K4 mono-methyltransferase MLL4 is targeted to active promoters via binding of its PHD6 domain to nucleosomes marked by H4K16ac [28]. Radzisheuskaya *et al.* recently showed that the same acetyltransferase, KAT8, catalyzes acetylation on distinct H4 residues (H4K5/8ac or H4K16ac) in different genomic regions depending on the complex it resides in [29]. It remains to be shown whether the specific recruitment of the complexes may depend on different chromatin states and modification patterns.

Specific combinations of histone modifications have been suggested to dictate context-specific functional outcomes. Bivalent chromatin regions are characterized by the co-localization of activating (H3K4me3) and repressive (H3K27me3) histone modifications, which poise genomic loci for subsequent activation or repression during development and these are often deregulated in cancer cells [10,30]. Similarly, the combination of H3K4me1 and H3K27ac is associated with active enhancers, while chromatin marked with only H3K4me1 is thought to indicate primed enhancers [31,32]. Interestingly, reduced expression levels of the methyltranferase MLL4, caused by KMT2D truncating mutations, not only impairs H3K4me1 deposition but also results in decreased levels of H3K27ac, due to changes in MLL4-dependent and Polycomb-dependent chromatin compartmentalization [33^•^]. Specific groups of genes may also be marked with combinatorial epigenetic patterns, facilitating their concordant and precise expression. Indeed, immediate early genes in developing sensory neurons possess a unique bipartite chromatin signature, comprising of active H3K27ac on promoters but repressive H3K27me3 on gene bodies, allowing for fast stimulus-dependent expression [34].

Different epigenetic pathways drive opposing outcomes, and maintaining a proper balance is critical for chromatin homeostasis. One prominent example is the ability of H3K36me2 to restrict PRC2-mediated deposition of the repressive mark H3K27me3 [35,36]. Interestingly, oncohistone mutations in H3 lysine 27 or lysine 36 (H3-K27M or H3-K36M, respectively), may alter this balance by affecting both the spread and levels of these modifications [37, 38, 39, 40, 41]. Similarly, loss of H3K27 methylation, due to impaired Polycomb activity results in hyperacetylation of this lysine residue [42], and prolonged deacetylation of H3K27 by inhibition of P300/CBP provides nucleation sites for its methylation to facilitate stable transcriptional suppression [43^•^]. Of note, the dependency of tumor cells on persistent oncogene expression can be therapeutically targeted by concomitant inhibition of both P300/CBP acetyltransferases and KDM6A demethylases [43^•^]. Overall, the above examples highlight the importance of elucidating the combinatorial chromatin language, and how it is altered during tumorigenesis.

## High-resolution methods to profile the combinatorial epigenome

**Figure 2:**
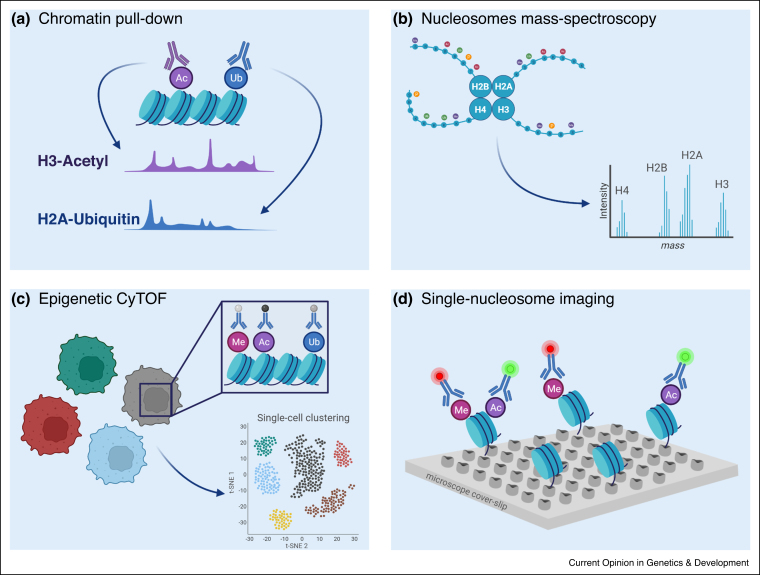
Novel methodologies to study the combinatorial epigenome. **(a)** Multiplexed pull-down based assays (ChIP-seq, Cut&Run and Cut&Tag) facilitate the mapping of combinations of marks to specific genomic locations. **(b)** Mass-spectroscopy based assays allow detection of multiple modifications originating from the same histone protein or nucleosome complex. **(c)** CyTOF enables global quantification of up to 40 different chromatin modifications at single-cell resolution. **(d)** Single-molecule imaging by TIRF microscopy reveals combinatorial patterns of histone modifications and oncohistones on millions of individual nucleosomes.

Better understanding of the combinatorial epigenetic landscape and its functionality requires multi-layered information that originates from complementary methodologies (Figure 2). The genomic localization of histone modifications is commonly analyzed by chromatin immunoprecipitation and sequencing (ChIP-seq), with recently developed methods such as Cut&Run and Cut&Tag providing higher resolution data from low-input samples and even single cells [44, 45, 46]. Yet, the combinatorial capacity of these methods remains limited; while pull down assays can identify the genomic location of a specific modification, they cannot effectively distinguish whether coincident marks coexist on the same nucleosome, or originate from different alleles or even different cells (when bulk analysis is performed). Adapting the Cut&Run strategy to utilize antibody barcoding via oligonucleotide conjugates, allows for simultaneous profiling of multiple histone modifications and chromatin regulators in individual cells, generating new tools to study the histone code [47^••^,^48^].

Mass-spectrometry (MS) can complement the sequencing-based methods, with its inherent capacity to detect numerous post-translational modifications on histone peptides, albeit without their genomic location [49]. While standard MS protocols remove the linkage between modifications and their nucleosomes of origin, Schachner *et al.* recently developed Nuc-MS, a methodology that captures the protein composition of native nucleosomes. This strategy reveals the co-occurrence of different histone variants and modifications within the same nucleosome structure, and is independent of antibodies [50^••^].

While MS provides combinatorial information at the nucleosome or histone peptide level, it relies on pooling material from a large number of cells. The Cytometry by Time of Flight (CyTOF) methodology allows highly multiplexed analysis of up to 40 different histone modifications and regulators in single-cells, albeit at low resolution (measurements represent global levels of each mark per cell) [51]. The single-cell nature of this assay reveals epigenetic heterogeneity in populations of cells and between individuals, which has been understudied. By applying CyTOF to blood immune cells, recent studies showed increased epigenetic heterogeneity associated with ageing [52] and following vaccination [53^•^]. Similar high dimensionality profiling of pediatric gliomas driven by H3-K27M revealed two distinct epigenetic subpopulations, driven by the expression levels of the oncohistone, with functional consequences to the tumor biology [54].

Imaging-based approaches that combine spatial information with genomic loci, using DNA or RNA fluorescence *in situ* hybridization (FISH), facilitate our ability to identify functional interactions between modifications and the organization of chromatin within the nucleus [55, 56, 57]. Takei *et al.* integrated imaging of multiple chromosomal loci, along with sequential immunofluorescence of chromatin marks and RNA FISH. With this multi-dimensional dataset the authors detect spatial nuclear zones that depend on combinations of histone marks, and evaluate their heterogeneity between different cells [58^••^]. We developed a single-molecule based approach to image individual nucleosomes and decode their combinatorial epigenetic states, followed by single-molecule DNA sequencing to reveal their genomic localization [59]. Furth *et al.* applied it to tackle fundamental questions relating to chromatin incorporation of H3-K27M, and identified enrichment of H3K4me3 on these oncohistones [40]. Additional single-molecule based approaches, using force spectroscopy and live cell imaging, provide important mechanistic understanding of the forces underlying the binding and dynamics of chromatin regulators, such as PRC2 [60,61]. We expect these novel methodologies to profoundly impact our understanding of epigenetic regulation of genome function.

## Combinatorial epigenetics as potential markers for cancer diagnostics

Recent advances in non-invasive liquid biopsy methods, based on the analysis of cell-free DNA (cfDNA), potentiate new generation approaches for cancer diagnostic and monitoring. The cfDNA that circulates in the plasma and serum of healthy individuals originates from death of normal blood cells [62]. In cancer patients, however, a fraction of cfDNA is tumor-derived, termed circulating tumor DNA (ctDNA). Analysis of ctDNA reveals tumor-specific genetic alterations providing the means for non-invasive molecular profiling of tumors [63,64]. Importantly, ctDNA also contains rich epigenetic information, including both DNA and histone modifications, which may increase the accuracy of detection and indicate the tumor tissue-of-origin [65,66]. A prominent example is DNA methylation, which regulates tissue-specific gene expression, and thus can be used in order to match ctDNA to the tumor site. Indeed, recent studies show that methylation at specific DNA loci can serve as a marker of tissue-specific cell death [67, 68, 69].

Similar to DNA, histone modification patterns reflect the epigenetic state of the tissue-of-origin and provide an additional layer of information in liquid biopsy samples. Nevertheless, extracting this type of information remains technically challenging. This is mainly due to the extremely low amount of tumor nucleosomes present in the blood. Sadeh *et al.* applied ChIP-seq for active chromatin modifications on plasma samples, showing it can be used to deduce expression programs and identify the cell-of-origin [70^•^]. Recent work by Fedyuk *et al.* applied single-molecule imaging to probe a large panel of epigenetic modifications in plasma nucleosomes, as well as ratios between different marks and their combinatorial patterns, to diagnose colorectal cancer at high accuracy [71]. Such methodologies have high potential for diagnosing a wide spectrum of diseases, as well as for addressing basic questions of tumor epigenetic dynamics.

In conclusion, decoding the combinatorial and intertwined nature of the epigenetic language requires complementary experimental approaches that will capture and integrate different layers of information. This holistic approach is key to better understand how chromatin dynamics affects genome function and drives tumorigenesis.

## Conflict of interest statement

Nothing declared.

## References and recommended reading

Papers of particular interest, published within the period of review, have been highlighted as:

• of special interest
•• of outstanding interest
